# Substrate wettability guided oriented self assembly of Janus particles

**DOI:** 10.1038/s41598-020-80760-w

**Published:** 2021-01-13

**Authors:** Meneka Banik, Shaili Sett, Chirodeep Bakli, Arup Kumar Raychaudhuri, Suman Chakraborty, Rabibrata Mukherjee

**Affiliations:** 1 Instability and Soft Patterning Laboratory, Department of Chemical Engineering, Indian Institute of Technology Kharagpur, Kharagpur, 721302 India; 2 S. N. Bose National Centre for Basic Sciences, J D Block, Sector III, Salt Lake City, Kolkata, 700106 India; 3 School of Energy Science and Engineering, Indian Institute of Technology Kharagpur, Kharagpur, West Bengal 721302 India; 4 Department of Mechanical Engineering, Indian Institute of Technology Kharagpur, Kharagpur, West Bengal 721302 India

**Keywords:** Colloids, Self-assembly

## Abstract

Self-assembly of Janus particles with spatial inhomogeneous properties is of fundamental importance in diverse areas of sciences and has been extensively observed as a favorably functionalized fluidic interface or in a dilute solution. Interestingly, the unique and non-trivial role of surface wettability on oriented self-assembly of Janus particles has remained largely unexplored. Here, the exclusive role of substrate wettability in directing the orientation of amphiphilic metal-polymer Bifacial spherical Janus particles, obtained by topo-selective metal deposition on colloidal Polymestyere (PS) particles, is explored by drop casting a dilute dispersion of the Janus colloids. While all particles orient with their polymeric (hydrophobic) and metallic (hydrophilic) sides facing upwards on hydrophilic and hydrophobic substrates respectively, they exhibit random orientation on a neutral substrate. The substrate wettability guided orientation of the Janus particles is captured using molecular dynamic simulation, which highlights that the arrangement of water molecules and their local densities near the substrate guide the specific orientation. Finally, it is shown that by spin coating it becomes possible to create a hexagonal close-packed array of the Janus colloids with specific orientation on differential wettability substrates. The results reported here open up new possibilities of substrate-wettability driven functional coatings of Janus particles, which has hitherto remained unexplored.

## Introduction

Ever since the Nobel award lecture by de Gennes^1^, Janus colloids with two or more chemically distinct domains have been extensively used as anisotropic building blocks for creating uniquely organized hierarchical nanostructures^2,3^, due to distorted rotational symmetry arising out of dipolar or chemical anisotropy which results in orientation-dependent interactions. While one of the first applications of Janus particles was in enhancing stability of oil water emulsions^4^, these have also been used as solid surfactants and optical probes for measuring rotational diffusion^5^. Orientation dependent interactions in Janus particles leads to rich phase behavior due to non-ideal translation and rotation^6,7^, and consequently forms aggregates that are rich in both dynamics and morphology^8,9^, as compared to their homogeneous counterparts. Formation of many distinct and unique assemblies with Janus particles has been reported in recent years. For example, by accurately controlling the Janus balance of triblock Janus particles, Granick and co-workers showed that Janus colloids can self-assemble into complex predetermined crystal structures such as 2D Kagome lattice^10–12^, which offers enhanced functionality due to the presence of alternate hydrophobic and hydrophilic pores in the structure.

Interestingly in most cases,the self-assembly and ordering of Janus particles have been achieved either in a liquid medium or at a liquid–air interface^7,13^, and there is limited literature on the influence of a solid surface on the orientation and ordering of these particles. By drop-casting gold (AuC8) nanoparticles partially covered with 3-mercapto-1,2-propanediol (MPD) from a dilute solution in water, Xu et al. showed that the domain sizes were larger with Janus particles than the homogeneous particles, which was attributed to the asymmetrical particle–surface interaction that led to super-particulate assemblies, similar to conventional surfactant molecules^14^. However, no preferred orientation of ordering of the particles was observed. Recently, Miller et al. have shown that amphiphilic Janus particles assemble with their hydrophobic faces oriented upwards upon drying over a hydrophilic surface, though the aggregates were in the form of fractals and lacked any long-range order^15^. Yang et al. showed the possibility of obtaining HCP array with Janus particles by seed-directed electrophoretic deposition^16^, though it is limited to a rather small area. Apart from addressing fundamental questions associated with surface-directed assembly of Janus particles, such ordering may have major practical relevance, motivated by the fact that two-dimensional (2D) hexagonal close-packed (HCP) arrays with homogeneous colloids are extensively used as optical chips^17^, photonic bandgap materials^18^, data storage^19^, sensors^20^, masks for nanosphere lithography^21^, and so on. The only practical route by which arrays of Janus particles is fabricated is by depositing a metal layer on a pre-existing ordered monolayer colloidal array by topo-selective glancing angle deposition (GLAD) or other deposition techniques using appropriate shadow masks^22,23^. Such an approach is widely used for making active matter particles such as micro swimmers^24^. However, this approach does not bring out the precise role of the substrate wettability in guiding the assembly of Janus particles on the substrate itself.

Here, we show the unique role of substrate wettability in directing the orientation of an amphiphilic metal-polymer Janus particle drop cast over a solid, homogeneous substrate. We experimentally show that on a hydrophobic substrate, the hydrophilic (metallic) face of all the particles orient outward. In contrast, on a hydrophilic substrate, the hydrophobic (polymeric) face of the particles orients outwards. No preferred orientation is observed on a substrate with intermediate wettability. These results are further substantiated by molecular dynamic simulations, which showcase that the ordering and density distribution of water molecules near the particle substrate interface leads to an alteration in the net driving force on the particles due to change in substrate wettability, which in turn controls the orientation of the deposited particles. Finally, we show that by spin coating, it becomes possible to create large area HCP array of Janus particle on different wettability substrates, where the orientation of the particles is determined by the substrate wettability. This is attributed to the coupling of wettability dependent substrate–particle interaction with capillary force mediated ordering of the particles during their self-organization and formation of ordered monolayer 2-D crystalline domains^25^. To the best of our knowledge, this work reports the first experimental evidence where an array of Janus particles with perfect orientational and spatial ordering has formed on a solid substrate with tailored wettability following a solution processed route.

## Molecular simulation

**Figure 1:**
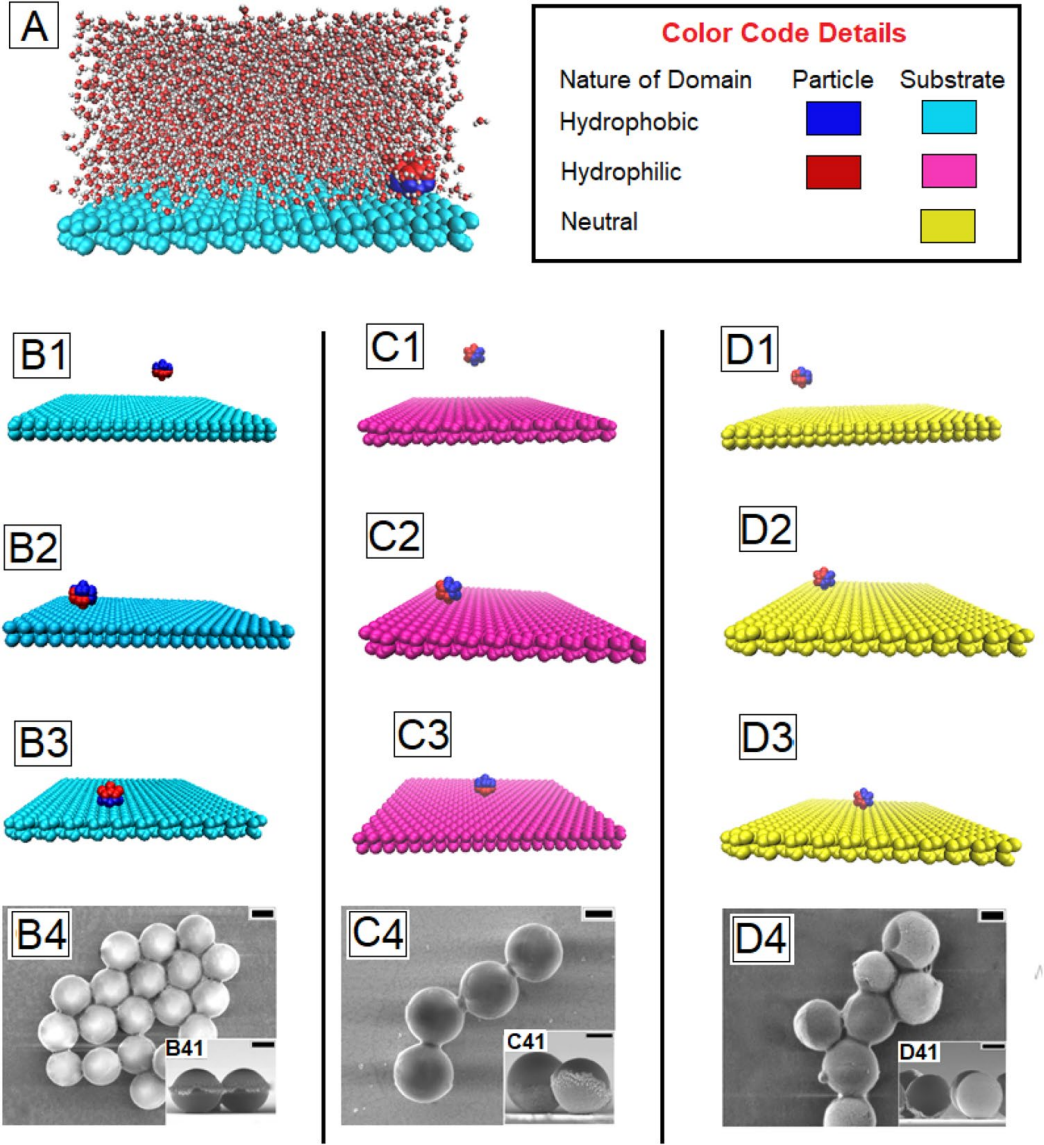
(**A**) **S**napshot of the system studied, comprising Janus particles in a semi infinite pool of water over a substrate or wall. The wettability is represented by the color coding for ease of viewing. (**B**–**D**) Simulation snapshots of the evolution of orientation of a single Janus particle as it approaches a non-wettable, a wettable, and a neutral substrate respectively, starting from an initial random orientation. To facilitate visualization, the water molecules are made invisible. The three frames in each column correspond to the snaps taken at 0 ns, 50 ns and 180 ns of the simulation run respectively. The third snapshot in each column (**B3**, **C3** and **D3**) refers to the orientation of the particles after it has reached the substrate. The last images in each column (**B4**, **C4** and **D4**) are FESEM images of the actual particles deposits after the solvent has fully evaporated away. The insets to these frames show the cross-sectional image to depict the orientation of the particles. The scale bar in each frame is 300 nm.

In order to obtain a theoretical perspective about the underlying physical phenomenon that leads to oriented deposition of the Janus particles on substrates with different wettability, molecular dynamics (MD) simulations were excuted. Despite the apparent disparities in the experimentally and theoretically accessible physical scales, the potential success of the MD simulations in mimicking the experimental trends stems from the adequacy of the theoretical framework in capturing two distinctive levels of interfacial inhomogeneity. On a first level, this is exhibited by the heterogeneities of the two faces, and on a second level, it is manifested by the heterogeneity at the Janus boundary that is chemical as well as topologically driven depending on the exclusive details of the particle functionalization. In an effort to mimic this essential physics of interest, our MD simulation platform considered particles suspended in a bath of a semi-infinite pool of water, as shown in Fig. 1A. The substrate was modeled using four layers of atoms in Face Centered Cubic (FCC) lattice in < 100 > plane. Each unit cell had lateral dimensions of 50 × 44 units and the height of water layer was taken to be 29 units (9.2 nm) above the free surface. Periodic boundary conditions were applied in the ***x*** (axial) and ***y*** (transverse) directions. The number of water molecules in the channel conformed to the bulk density of water at 300 K. The wall atoms interacted via Lennard Jones (LJ) potential and water molecules conformed Simple Point Charge/Extended (SPC/E) model^26^. Each particle was formed by enclosing all atoms within a radius of 6 units from a central atom,with FCC lattice structure^27,28^. To test the size dependence, particles having radii of 4 and 8 units were also considered (shown in SI). However, there was no appreciable deviation in the density distribution of the surrounding water molecules or temporal evolution of the particle orientation as a function of the size of the particles. The resulting particle had a rough structure, diametrically segregated into two distinct domains that were tuned to have high heteronuclear attraction potential between them. This choice of potential allowed the domains to stick to each other, retaining the spherical shape, mimicking the actual Janus particles. The heteronuclear interaction of each domain with water and wall molecules further tuned via an adjustable Lennard–Jones (LJ) potential: $$V_{LJ} \left( r \right) = 4\varepsilon \left[ {\left( {\frac{r}{\sigma }} \right)^{ - 12} - \left( {\frac{r}{\sigma }} \right)^{ - 6} } \right]$$, involving an atomic length scale σ, energy scale . The relevant time-scale for an atomic mass of m turns out to be $$\tau = \sigma \left( {{\raise0.7ex\hbox{$m$} \!\mathord{\left/ {\vphantom {m \varepsilon }}\right.\kern-\nulldelimiterspace} \!\lower0.7ex\hbox{$\varepsilon $}}} \right)^{{{\raise0.7ex\hbox{$1$} \!\mathord{\left/ {\vphantom {1 2}}\right.\kern-\nulldelimiterspace} \!\lower0.7ex\hbox{$2$}}}}$$. The wall atoms were thermostated at a fixed temperature of $$0.8\varepsilon /k_{B}$$, $$k_{B}$$ being the Boltzmann constant, using local Nosé-Hoover thermostat and the fluid molecules dissipated through the flexible wall atoms. The long-range electrostatic interactions were obtained using Particle Ewald Mesh (PME) method. The system was energy minimized and then equilibrated using leap-frog algorithm for 1000 time units ($$10^{6}$$ time steps). Following this, the system was studied using equilibration run for 500,000 time units, integrated using leap-frog algorithm with a step size of 0.001 units or 500 ns. The time scale was normalized considering $$\varepsilon \sim 48k_{B} T$$.The MD simulation platform conformed to an NVT ensemble, consistent with the standard procedure.

All the corresponding interaction parameters used in our simulation are given in Table 1, W and P refer to the wall and a particle, respectively. The subscripts hb/hp/n with the wall or substrate denote hydrophobic/hydrophilic/neutral interactions, respectively. The subscripts nw/w indicate non-wetting and wetting domains of the particle, respectively. All the rows denote homonuclear interactions, with mixing rules describing the non-bonded interactions, except for the last row which gives the heteronuclear interaction between the two domains. The pair-potential between the wetting and non-wetting domains is explicitly defined to maintain the stability of the Janus particle.The interaction potentials are determined from the choice of the experimental materials for which the static contact angles are first experimentally obtained. Using the values of these contact angles, we reverse-map simulated flat surfaces to obtain the Lennard–Jones (LJ) parameters, based on curve-fitting of the tabulated data of these parameters as a function of the contact angle, mimicking the experimental condition.

**Table 1:** LJ interaction parameters for different particles.

Interaction	ε (kJ/mol)	σ (Å)
H–H	0	0
O–O	0.650	3.1656
W_hb_ − W_hb_	0.8	3.40
W_hp_ − W_hp_	5.9	3.40
W_n_ − W_n_	1.9	3.40
P_nw_ − P_nw_	1.1	3.40
P_w_ − P_w_	5.3	3.40
P_w_ − P_nw_	6.5	3.40

The physics of particle–substrate-fluid interaction is essentially governed by the energetics of the system. The interfacial free energy is obtained from an integral of the appropriate local free energy per unit area, alternatively designated as the surface tension. The free energy of a Janus particle at an interface may be calculated by noting that for each orientation angle $\theta$ and depth $z$ in water, elemental interfacial energy can be obtained over a differential surface area element $dS\left( {\mathbf{r}} \right)$. From this, the free energy of a circular region of an interface of radius $r_{S}$ can be subtracted ($\gamma^{f}$ being the energy per unit area), so that the interfacial energy can be obtained as: $\Delta F = \int\limits_{IS} {dS\left( {\mathbf{r}} \right)} \left[ {\gamma_{SL} \left( {\mathbf{r}} \right) - \gamma_{SV} \left( {\mathbf{r}} \right)} \right] - \pi r_{S}^{2} \gamma^{f}$, where the integral runs over a spherical immersed surface (IS). The difference in interfacial energies of solid–liquid and solid-vapour combination, as appearing in the integrand, either corresponds to the non-wetting or the wetting face of the Janus particle, depending on whether the non-wetting or the wetting phase is touching the polar liquid phase (water). The solid surface energy differences can be calculated by thermodynamic integration method^28^, in which a path is selected for the configuration of the particle and the position of its center. At each configuration, the particle is fixed. First, the system is allowed to equilibrate and subsequently, the force on the particle is measured as time-average. As the temperature is constant along the path, the change in free energy between any two configurations corresponds to the work done to commute the particle reversibly between them. Modulations in this free energy essentially turn out to hold the key towards dictating the preferential orientation of the particle, as demonstrated subsequently.

## Results and discussions

The first three images of series B to D of Fig. 1 show the reorientation of the Janus particles as they approach a non-wettable, wettable and neutral substrate respectively, starting from a random initial configuration, based on MD simulations. The last image in each series is an FESEM of the particles deposited on the respective substrates, after complete evaporation of water. It can be seen from the tilted cross-sectional images shown as inset of Fig. 1B4 that, on a hydrophobic surface, the metallic or the wettable side of all the Janus particles face outwards, so that the hydrophobic (polymeric) face of the Janus particle is in contact with the hydrophobic substrate. An exact opposite orientation of the particles is observed on a hydrophilic substrate, where the metallic faces are seen to be in contact with the substrate (inset C41 of frame C4, Fig. 1). Inset D41 of frame D4, Fig. 1 reveals that the particles are orientaed randomly on a substrate with intermediate wettability. It can be seen that frames B3, C3 and D3 capture the exact same orientation that is observed in actual experiments, highlighting perfect agreement between the simulations and experimental results.

**Figure 2:**
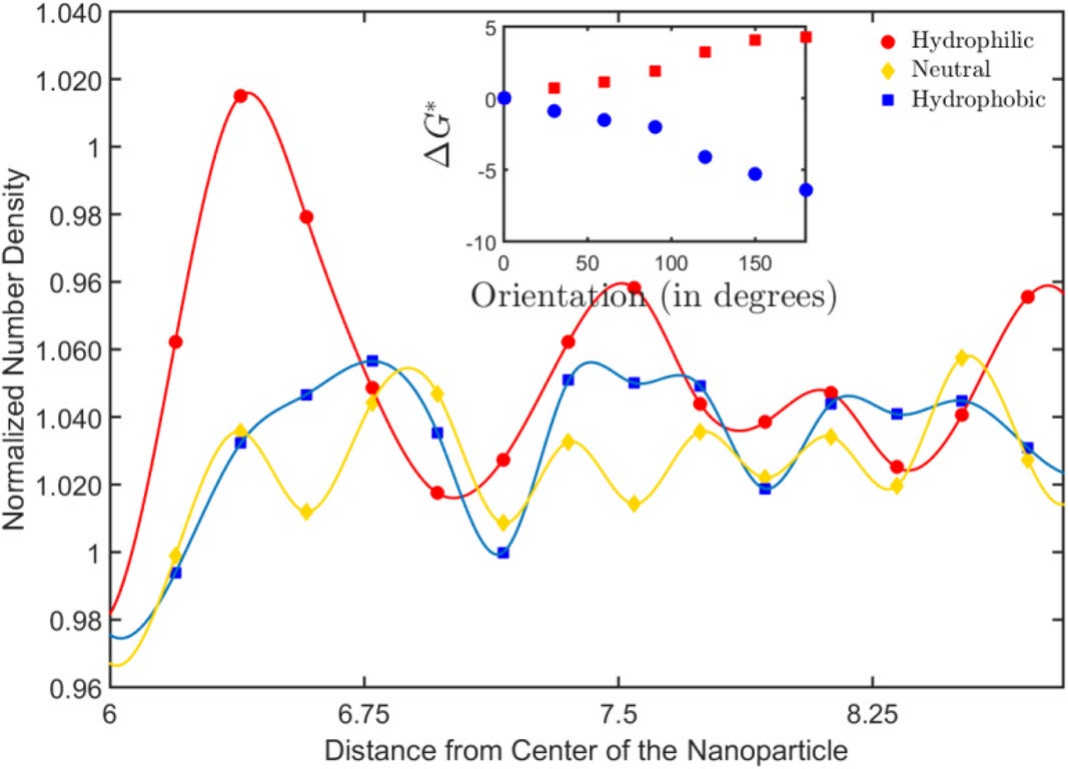
Number density variation of water molecule near particles having surfaces of different wettabilities. The water density crests and throughs alter the energy landscape around the particle, and in the process, favor particular orientation as also observed with the free energy calculations for reorientation, as shown in the inset.

The dextrous reorientation of the Janus particles as they reach the fluid substrate interface can be understood from the molecular arrangement of water molecules near surfaces of different wettabilities. It is well known that water molecules near a hydrophilic surface not only exhibit long-range ordering but also lead to significant enhancement of local density as compared to the bulk density. On the other hand, hydrophobicity induces a density depletion zone with the depth of the zone depending on the degree of hydrophobicity^29,30^. Therefore, the density distribution of water molecules near a hydrophilic surface results in a higher magnitude of cohesive energy as compared to the density distribution around a hydrophobic surface.The number density distribution of water near a hydrophobic, hydrophilic and neutral surface of a particle is shown in Fig. 2. By including the thermal fluctuations along with this description, the Boltzmann distribution can be used to predict the evolution of the system. With the two states of density segregated water molecules around wetting and non-wetting surfaces, the equilibrium would be maintained with $\mu_{hpl}^{i} + TS_{hpl} = \mu_{hpb}^{i} + TS_{hpb}$ where $\mu_{hpl}^{i} \,/\mu_{hpb}^{i}$ is the chemical potential in the hydrophilic/hydrophobic region and ***S*** with the specific subscripts represent the entropy of solution. Thus, we have entropy induced enrichment near the hydrophilic face and a depletion near hydrophobic face, over and above the density distribution determined by the wettability. This transient redistribution is only significant when the particle is near the interface and not in the bulk. As the Janus particles approach the surface, the zone around the particle has four different density distributions: the density augmentation/depletion next to the surface, the density augmentation and depletion across the diameter of the Janus particle and the bulk density. The total surface free energy of the particle can be expressed in the in terms of the surface tension coefficients between the faces of the particle and the surrounding fluid, as delineated earlier. We use subscripts ‘A’ for the hydrophobic/apolar face and ‘P’ for the hydrophilic/polar face. The density enriched/depleted/bulk phases of water are denoted by subscripts ‘E’, ‘D’ and ‘B’, respectively. The total surface energy can be expressed in terms of the orientation of the particle as^31^,

1 $$E = 2\pi \hbox{R}^{2} \left[ {\gamma_{AD} \left( {1 + \cos \beta } \right) - \gamma_{AB} \cos \beta + \gamma_{PB} - \frac{1}{2}\gamma_{DB} \left( {\sin^{2} \beta } \right)} \right]$$

where R is the radius of the particle and $\beta$ is the immersion depth denoting the orientation of the faces of the Janus particle in the density depleted and bulk density phases of water distribution parametrized in terms of angle. In this example,$\beta > 90^{ \circ }$ due to the inverted initial configuration, which is finally replaced by the “heads-down” assembly at the interface, with the total free energy given by

2 $$E = 2\pi \hbox{R}^{2} \left[ {\gamma_{AD} + \gamma_{PB} - \frac{1}{2}\gamma_{DB} } \right]$$

The above gives the minimum energy configuration. The solvation interactions aid the entropic interactions with a wettable substrate leading to faster reorientation dynamics for a hydrophilic substrate, as observed in MD simulations, compared to a hydrophobic substrate.

Using the simulation results, we obtain the difference in free energy for different configurations with the particle sedimented on the substrate. To obtain this, we assume a base configuration, where the equatorial plane of the Janus particle is aligned parallel to the surface with the wetting hemisphere facing the substrate. Any rotation of the equatorial plane is denoted in terms of angles, with 0° representing the wetting face downwards configuration and 180° denoting the non-wetting face down configuration. The values of the differences in free energy are normalized by the thermal energy $k_{B} T$ for ease of representation. The free energy data for different configurations of the particle on wettable and non-wettable surfaces, as seen inthe inset of Fig. 2, attests to the physics behind reorientation described from the density fluctuations observations described above.

Interestingly, on a surface of intermediate wettability with a contact angle around 50°_,_ the final orientation turns out to be independent of the wettability and dependent on the initial orientation (series D, Fig. 1). This can be explained by lower total surface energy for a random configuration with and acute $\beta$ as compared to the “heads-down” or “heads-up” configuration. The interfacial water in this case has neither depletion nor enhancement of number density. Hence, there is no longer a stark contrast between the surface tension of bulk water and interfacial water. The particle after approaching the interface does not have a particular orientation which would minimize the energy. As a result, the thermal motion of the water molecules drives the particle along the interface and the reorientation of density enriched and density depleted water across the diameter of the particle may provide nudge in either direction. However, once the particle is at the interface, it is rarely observed to travel back to bulk, as the substrate being weakly wetting has more affinity towards the particle and the subsequent motion of the particle is along the plane of the substrate.

**Figure 3:**
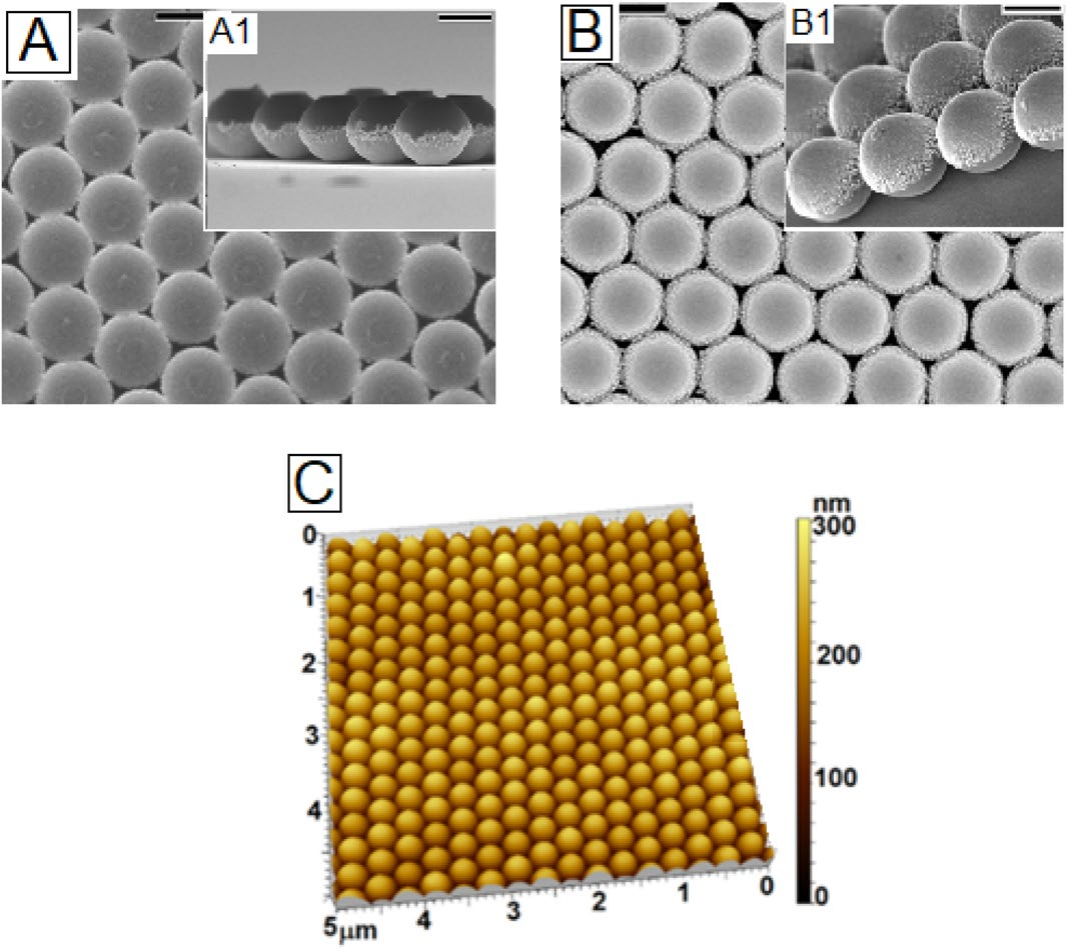
Top-view FESEM image of the spin coated Janus particles (dia 300 nm) on (**A**) gold coated silicon wafer (hydrophilic) and (**B**) silanized silicon wafer (hydrophopic). Insets A1 and B1 show the respective tilted images, which capture the orientationof the particles. (**C**) AFM image corresponding to the FESEM image shown in frame A.

Once the role of the substrate surface energy in determining the orientation of the Janus particle deposited on it from a solution was fully understood, we tried to create large area monolayer HCP array with the Janus particle on substrates with different wettabilities by spin coating, a method that has been widely used for creating colloidal array with homogeneous particles due to ease of execution and requirement of very low amount of colloidal dispersion^32^. By spin coating, we could obtain perfectly ordered HCP patches with the Janus particles over both hydrophilic (Fig. 3A) and hydrophobic (Fig. 3B) and substrates respectively. Spin coating was performed by rotating the substrate at 800 RPM for 2 min duration^32^. The AFM image in Fig. 3C shows the large area defect free nature of the HCP domains obtained on the hydrophilic substrates. In fact we find, similar to that observed by Xu et al.^14^, that the sizes of the defect-free domains are larger with the Janus particles. This can be attributed to supreesion of dewetting during capillary driven re-organization of the particles at the array formation stage, as observed in-situ for a low concentration low evaporating solvent by Howse and co-workers^25^. As the preferred end of the Janus particles, in the present scenario, comes in contact with the substrate, it gets anchored and stops to roll. This significantly enhances the resistance against contact line retraction, limiting the size of the holes or defect domains, and increasing the size of the defect free pathces (refer to figure S4 of online supporting information). This clearly highlights that during spin coating mediated deposition, in addition to the structure and local density of the solvent molecules which are shown to govern the orinetation of the particles in case of drop casting, the presence of the Janus particles hinders the dewetting process by imposing jamming effect, similar to that observed in the context of thin film dewetting suprression with compatible particles^33^. The patches laterally spanned upto 100 μm^2^ area.Following our experience, the size of these defect free domains can be increased by careful optimisation of the coating conditions, which is beyond the scope of this paper, and will be taken up separately. We must admit that on neutral substrates we failed to obtain perfect close packed ordering despite trying various different spinning conditions.

Formation of ordered colloidal particles array by spin coating is a complex process, as it involves non-uniform unsteady flow. The most comprehensive theoretical framework till date, under which ordering of colloids can be achieved by spin coating has been proposed by Shereda et al.^34^ They predicted the possibility of high steady-state crystallization when:

$${\varvec{Pe}} = \user2{ }\frac{{{\varvec{\tau}}_{{{\varvec{rz}}}} {\varvec{a}}^{3} }}{{{\varvec{k}}_{{\varvec{b}}} {\varvec{T}}}} = \user2{ }\frac{{6\user2{\pi \rho \omega }^{2} {\varvec{r}}\left( {{\varvec{h}} - {\varvec{z}}} \right){\varvec{a}}^{3} }}{{{\varvec{k}}_{{\varvec{B}}} {\varvec{T}}}} > {\varvec{Pe}}_{{\varvec{c}}}$$

where ***Pe*** is Peclet Number, ***τ***_***rz***_ is shear stress, a is the diameter of the colloids, and ***k***_***b***_***T*** is the average translational Kinetic energy of the colloids, ***ρ*** is the density, ***ω*** is the rotational speed, ***r*** is the radial distance from the centre, ***h*** is the film thickness and ***z*** is the height from the substrate. It has been previously shown by Ackerson and Pusey that a steady or oscillatory flow favours the formation of colloidal crystal arrays with the hexagonal close-packed arrangement when ***Pe*** > O(1) (Order of unity)^35^. Shereda et al. attributed the formation of uniform colloidal crystals to the coupling of two stress-controlled physical processes. At the macroscopic level, spin coating is stress-controlled as the flow is entirely driven by centrifugal force and consequently local stress is a function of ***ω*** and ***h***. On the other hand, at the microscopic level flow induced colloidal crystallization is also stress-controlled as excessively high or extremely low stress hinders colloidal crystallization. It may be pointed out that the formulation by Shereda et al. does not take into consideration the effect of evaporation, which is a critical parameter that determines ***h ***in spin-coating and is therefore likely to strongly influence the crystallization process as well. For Janus particles, the situation is likely to become more complex as the forces arising out of the reorganization of the particles close to the substrate surface just ahead of deposition also augments with the forces responsible for the array formation. Till date, however there was no experimental evidence of the formation of a hexagonal close-packed array of Janus particles on a solid substrate by spin coating, and probably as a consequence of that, no theoretical formulations on the same have been developed. We must admit that within the framework of the present paper, it is also not possible to capture or simulate the array formation. While MD simulations here has been used as a tool to simulate the particle–surface and particle-solvent interactions at the various stages of array formations and orientation of Janus particles on the surface, using MD to determine particle–particle interaction in an evaporating system is computationally prohibitive not only due to a large number of substrate and solute particles needed to mimic the experiment but also lack of existing formulation or the varying solvent density mimicking evaporation in a rotating frame of reference and thus, remains a open question at least for the time being, which is worth looking into in the future^36^.

## Conclusions

In summary, we have shown, based on experiments as well as molecular dynamic simulations, that the orientation of an amphiphilic Janus particle dispensed on a solid substrate depends on the wettability of the substrate. While gold-PS Janus particle deposited in the head-down (gold face facing downwards) configuration on a hydrophilic substrate, the same particle oriented in the head-up configuration on a hydrophobic substrate. No preferred orientation of the particle was observed on a substrate with intermediate wettability. Simulations show that the orientation of the particle can be uniquely modulated by exclusive alterations in the interfacial free energy between the particles and the substrate. Variation in the substrate wettability of the substrate results in difference in the organization of the water molecules in proximal contact with the substrate. These results reveal that dextrous orientation of Janus particles is a macroscopic manifestation of balance between the solvation entropy around a heterogenous solute particle, which may be controlled by modulating the substrate wettability. Further, by spin coating the Janus particles, we could achieve perfectly ordered hexagonal closed packed array of the Janus colloids with orientation determined by the substrate wettability. Our findings are likely to be of importance in creating super functional colloidal coatings with Janus paticles and possible realisation of optofluidic mirrors or gaining intricate controls on the initial placement of array of catalytically driven self-propulsion of active colloidal particles.

## Methods

The Janus particles were created by well known toposelcetive deposition of Gold on a pre-existing array of polystyrene (PS) particles (dia 300 nm and 600 nm) obtained by spin coating on a poly(methyl methacrylate) (PMMA) coated substrate^37,38^. The particle array was first exposed to oxygen plasma in an Inductively Coupled Plasma Reactive Ion Etching (ICP-RIE) chamber for 6 s for slight reduction in size (the diameter of the colloids reduced to 285 nm and 583 nm respectively) so that the neighboring particles do not remain in contact during the subsequent metal deposition step. Post etching, around 10 nm thick gold layer was deposited on the PS particles in an electron beam evaporator.Subsequently, the Gold-PS Janus particles were detched from the PMMA substrate into water by Ultra-Violet-Ozone (UVO) mediated degradation of the PMMA layer, described in details elsewhere^32^. The dispersion of the Janus particle in water was taken in a micro syringe and drop cast on substrates of different wettability. In our experimnets, gold coated silicon wafer (equilibrium water contact angle, **θ**_**E**_ ≈ 2°), silanized silicon wafer (**θ**_**E**_ ≈ 110°) and solvent cleaned silicon wafer (**θ**_**E**_ ≈ 47°) were used as model hydrophilic, hydrophobic and neutral (intermediate wettability) substrates, respectively. For obtaining the array, the Janus particle dispersion was spin coated to obtain HCP colloidal array.

The morphology of the drop cast as well as spin coated Janus particles was investigated using a Field Emission Scanning Electron Microscope (FESEM) (JSM7610F, JEOL, Japan) and atomic force microscopy (AFM; Agilent Technologies, AFM 5100) in intermittent contact mode using a silicon nitride cantilever (PPP-NCL, Nanosensors Inc., USA).

## Supplementary Information

Supplementary Information.
