# Supplementary material for: Substrate wettability guided oriented self assembly of Janus particles
Source: Sci Rep. 2021 Jan 13;11:1182. doi: 10.1038/s41598-020-80760-w (PMC7807062; doi:10.1038/s41598-020-80760-w)
Supplement: Supplementary file 1 — Supplementary Information. [file 41598_2020_80760_MOESM1_ESM.doc]

**Supporting Information for Surface Directed Self Organization of metal-polymer Janus particles**

Meneka Banik,^1^ Shaili Sett,^2^ Chirodeep Bakli,^3^ Suman Chakraborty,^4^ Arup K. Raychaudhuri,^2^ and Rabibrata Mukherjee^1*^

^1^Instability and Soft Patterning Laboratory, Department of Chemical Engineering, Indian Institute of Technology Kharagpur, West Bengal, Pin 721302, India

^2^S. N. Bose National Centre for Basic Sciences, J D Block, Sector III, Salt Lake City, Kolkata, 106, India

^3^School of Energy Science and Engineering, Indian Institute of Technology Kharagpur, West Bengal, Pin 721302, India

4Department of Mechanical Engineering, Indian Institute of Technology Kharagpur, West Bengal, Pin 721302, India

Corresponding author: ^*^[rabibrata@che.iitkgp.ac.in](mailto:rabibrata@che.iitkgp.ac.in)

**Simulation details**


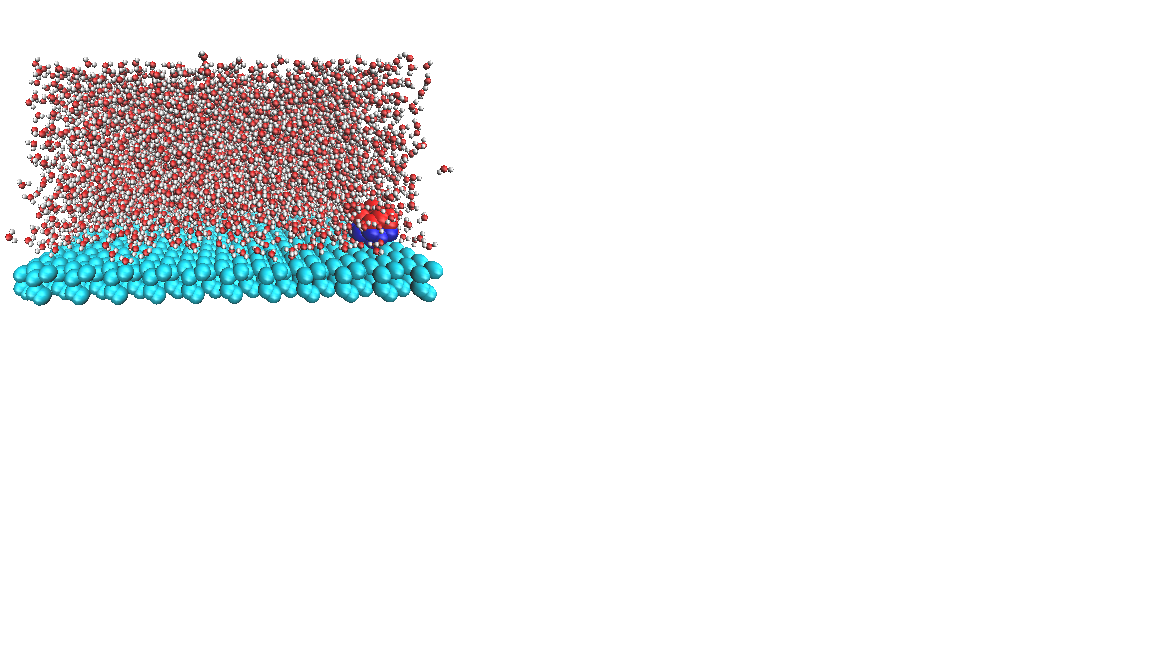


***Wetting Particles***

***Non-Wetting Particles***

***Neutral Particles***

**Figure S1.** *Simulation snapshot of the system studied.* *Water with nanoparticles was equilibrated on a wall with FCC structure. The wettability of the wall is tuned using LJ parameters to have a static contact angle of $120^{o}$. The* *nanoparticle is composed of hydrophobic and hydrophilic particles separated across the diameter forming a spherical structure with FCC lattice structure. The water molecules are added as per bulk density. The wettability is represented by the color coding for ease of viewing. Images A,B(1-3),C(1-3),D(1-3) are made with VMD 1.9.3 and is owned by the Theoretical and Computational Biophysics Group, NIH Center for Macromolecular Modeling and Bioinformatics, at the Beckman Institute, University of Illinois at Urbana-Champaign* [***http://www.ks.uiuc.edu/***](https://www.ks.uiuc.edu/)

The computational domain for MD simulations is chosen to mimic particles suspended in a bath of a semi-infinite pool of water. Fig. S1 depicts a typical simulation domain represented using a snapshot of a system with non-wetting walls. The substrate is modeled using four layers of atoms in FCC lattice in <100> plane. Each unit cell has lateral dimensions of $50\times44$units and the height of water is taken to be 29 units (9.2 nm) to the free surface. Periodic boundary conditions are applied in x (axial) and y (transverse) directions. The number of water molecules in the channel conforms to the bulk density of water at 300K Interactions for water molecules are defined using the Simple Point Charge/Extended (SPC/E) model ^1^. The LJ parameter $\sigma$for the wall atoms and the nanoparticle is taken to be the same as that for water molecules in SPC/E model. The nanoparticle is formed by a structure in FCC lattice formed by enclosing all atoms within a radius of $6$units from a central atom ^2,3^. We test for the size dependence of the nanoparticle on the observed behavior by using the radius of 4 and 8 units also. We do not observe any appreciable deviation in the density distribution of the surrounding water molecules or the temporal evolution of the nanoparticle orientation with variation in size. The resulting nanoparticle has a rough structure and we diametrically segregate into two kinds of particles. The density fluctuations for two different radii of particle are shown in fig. S2, which demonstrates the size-independence of density fluctuation and the resulting orientation dynamics.


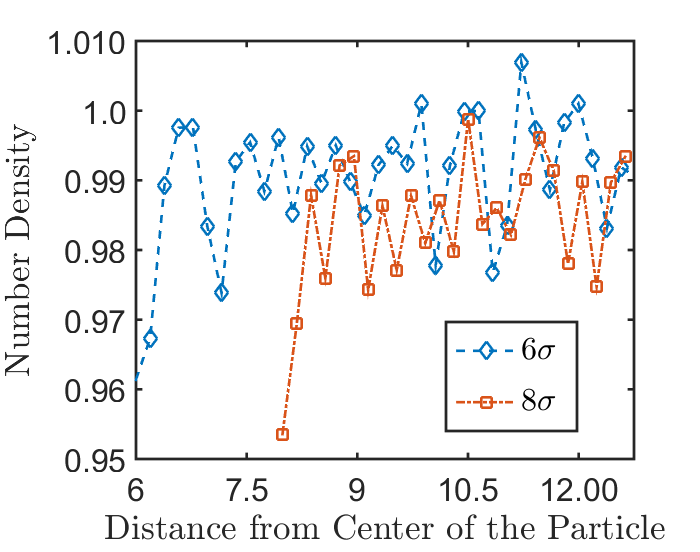


***Figure S2.*** *Dependence of density fluctuation on the size of Janus particle towards the non-wettable surface suspended in the bulk. The image was generated using MATLAB 2015a and the license was provided by http://www.cic.iitkgp.ac.in/*

These two types of particles are tuned to have high heteronuclear attraction potential among each other. This choice of potential helps the particles stick to each other, retaining the spherical shape. The heteronuclear interactions of each of these particle types with water and wall molecules are also tuned via LJ potential, where, having different heteronuclear $\varepsilon$gives the variation of wettability across the diameter and hence, simulating the chemical nature of a Janus particle. The wall atoms are thermostated using Nose-Hoover thermostat and the fluid molecules dissipate through the flexible wall atoms. The long-range electrostatic interactions are obtained using Particle Ewald Mesh (PME) method.

The system is energy minimized and then equilibrated using leap-frog algorithm for 1000 time units ($10^{6}$time steps). Following this, the system is studied using equilibration run for 500000 time units (integrated using leap-frog algorithm with a step size of 0.001 units or 500 ns) and the slip length is obtained from the resulting velocity profile. The time scale normalized using$\tau=\sqrt{\frac{m\sigma^{2}}{48k_{B}T}}$, where $m$is the mass of each molecule.

**Description of Reorientation Dynamics and Additional Results**

Combining the cohesive energy and the thermal fluctuations in the system, we can use the Boltzmann distribution to predict the evolution of the nanoparticle and the surrounding water molecules. With two states of density segregated water molecules around wetting and non-wetting surfaces, the equilibrium would be maintained with constant $\mu_{hpl}^{i}+TS_{hpl}=\mu_{hpb}^{i}+TS_{hpb}$, where $\mu_{hpl}^{i} /\mu_{hpb}^{i}$is the chemical potential in the hydrophilic/hydrophobic region and S is the entropy of the solution. Owing to entropic interactions, a recirculation of molecules would occur near the interfaces. From the density distribution obtained in MD simulations, we can predict that as opposed to a homogenous solute, a Janus particle would lead to the redistribution of water molecules from hydrophobic face to the hydrophilic face. Thus, we would have entropy-induced enrichment near the hydrophilic face and depletion near hydrophobic face, over and above the density distribution determined by the wettability. In other words, the density of water molecules near the wettable face of a Janus particle under equilibrium would be higher than that of the density of water molecules around a nanoparticle of the same dimension but uniformly hydrophilic. This transient redistribution of water molecules would not be of much consequence if the particle is suspended far away from the surface. However, this density alteration becomes significant as the particles tend to settle down on the surface. While in MD simulations, the particle settling down on the surface in a quiescent medium is strongly guided by the statistical fluctuations in the system; in a macroscopic system, the body force induced by gravity helps with the settling and indirectly with the reorientation process. In order to test our hypothesis, we applied a body force to our system along the direction of the substrate and as a result, the reorientation of Janus particles was much faster as compared to the data represented for equilibrium studies.

As the Janus particles approach the surface, the zone around the particle has four different density distributions; the density augmentation/depletion next to the surface, the density augmentation and depletion across the diameter of the Janus particle and the bulk density. We shall further illustrate this with an example.

Without loss of generality, let us consider the case shown in Fig 3 (a) in the main manuscript, i.e, a particle approaching a hydrophobic surface in a “heads-down” configuration. As it interacts with the surface there is a density depleted zone extending along the substrate, a locally enriched zone around the head which rams into this depleted zone, a more heavily depleted zone facing away from the surface and finally the bulk density. The total surface free energy of the particle can be expressed in the in terms of the surface tension coefficients between the faces of the particle and the surrounding fluid. We use subscripts ‘A’ for the hydrophobic/apolar face and ‘P’ for the hydrophilic/polar face. The density enriched/depleted/bulk phases of water are denoted by subscripts ‘E’, ‘D’ and ‘B’ , respectively. The total surface energy can be expressed in terms of the orientation of the particle as^41^

$$E=2\pi\mathrm{R}^{2}\left[\gamma_{AD}\left(1+\cos\beta\right)-\gamma_{AB}\cos\beta+\gamma_{PB}-\frac{1}{2}\gamma_{DB}\left(\sin^{2}\beta\right)\right]$$

where R is the radius of the particle and $\beta$is the immersion depth denoting the orientation of the faces of the Janus particle in the density-depleted and bulk-density phases of water distribution^18^. In this example, $\beta>90^{o}$due to the inverted initial configuration which is finally replaced by the “heads-down” assembly at the interface, with the total free energy given by:

$E=2\pi\mathrm{R}^{2}\left[\gamma_{AD}+\gamma_{PB}-\frac{1}{2}\gamma_{DB}\right]$ which gives the minimum energy configuration. The same can be demonstrated started with any random orientation and also for particles suspended near a hydrophilic surface. The only difference in the hydrophobic and hydrophilic substrate lies in the fact that the reorientation dynamics starts for a hydrophilic substrate at a greater height due to the long-range ordering as opposed to hydrophobic surfaces. The solvation interactions aid the entropic interactions with a wettable substrate, leading to faster reorientation time as observed in MD simulations compared to a hydrophobic substrate. In a hydrophobic substrate, the reorientation is majorly guided by the entropic redistribution of water molecules which imparts the force to reorient the particle in the density depleted zone. Interestingly, a third set of simulations on a surface of intermediate wettability with contact angle around 80^o^ demonstrated the final orientation to be independent of the wettability and dependent on the initial orientation (see figure S3). This can be explained by lower total surface energy for a random configuration with and acute $\beta$ as compared to the “heads-down” or “heads-up” configuration. The interfacial water, in this case, has neither depletion nor an enhancement of number density. Hence, there is no longer a stark contrast between the surface tension of bulk water and interfacial water. The particle, after approaching the interface, does not have a particular orientation which would minimize the energy. As a result, the thermal motion of the water molecules drives the particle along the interface and the reorientation of density-enriched and density-depleted water across the diameter of the particle may provide nudge in either direction. However, once the particle is at the interface, it is rarely observed to travel back to bulk, as the substrate being weakly wetting has more affinity towards the particle and the subsequent motion of the particle is along the plane of the substrate.

**
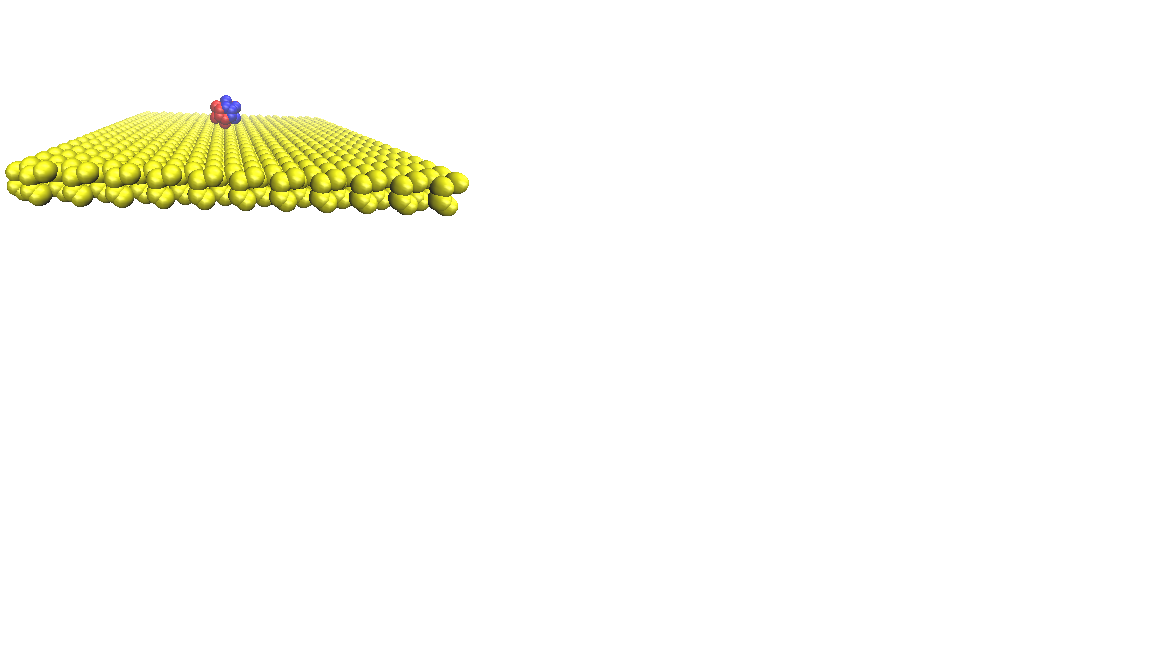

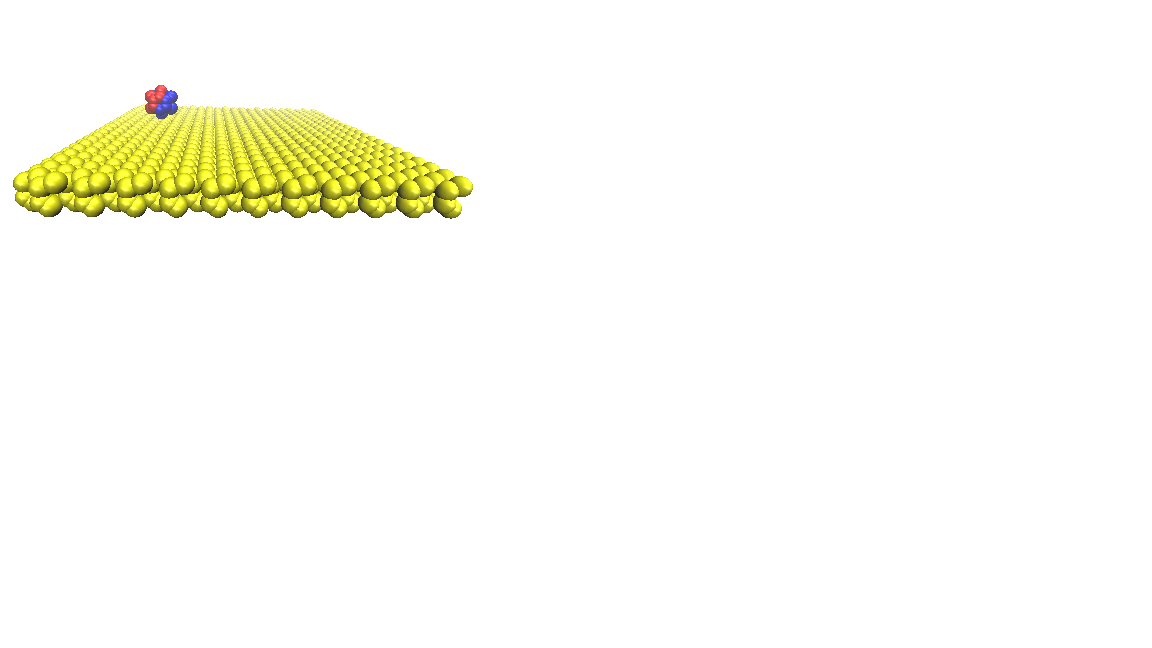

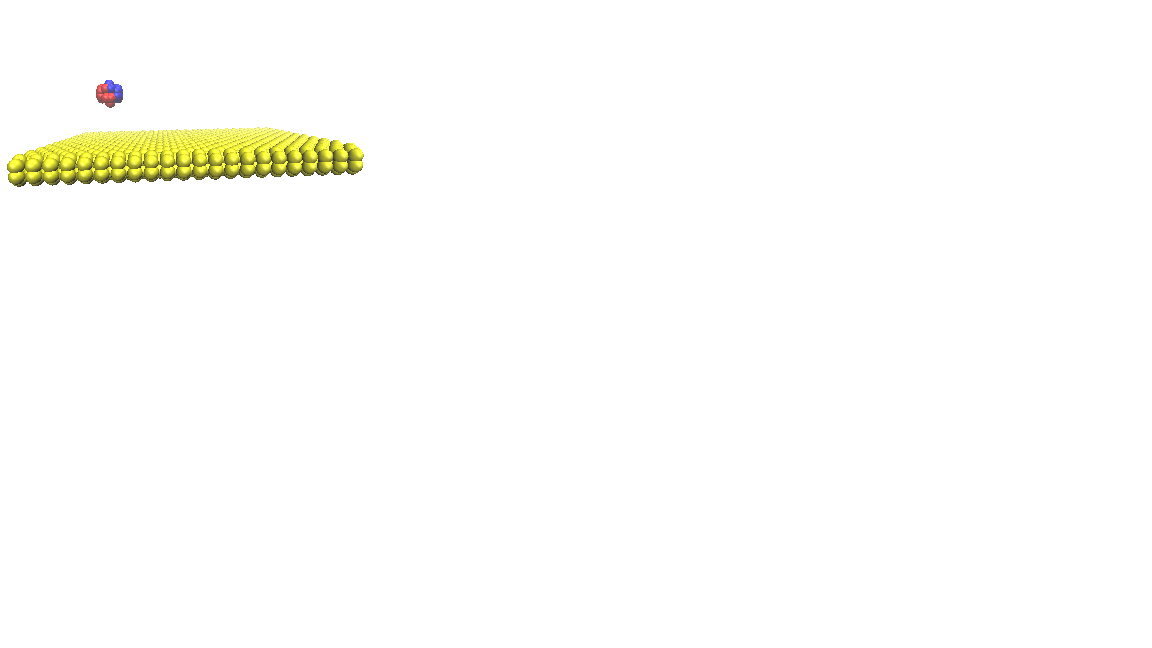
**

(a)

(b)

(c)

Figure S3. *Simulation snapshots of the evolution of orientation of a single Janus particle as it approaches a neutral substrate. To visualize clearly, we select one random Janus particle and the water molecules are made invisible. The simulation snapshots (a)-(c) depict a hydrophobic substrate with an equilibrium contact angle of $80^{o}$with the snaps taken at 0 ns, 60 ns and 180 ns of the simulation run respectively. Irrespective of being near to the interface, the particle does not reach a minimum energy for any configuration and hence we observe no particular steady state orientation and the particle keeps flipping due to thermal fluctuations. Images are made with VMD 1.9.3 and is owned by the Theoretical and Computational Biophysics Group, NIH Center for Macromolecular Modeling and Bioinformatics, at the Beckman Institute, University of Illinois at Urbana-Champaign* [***http://www.ks.uiuc.edu/***](https://www.ks.uiuc.edu/)

**Experimental visualization of the coated arrays**


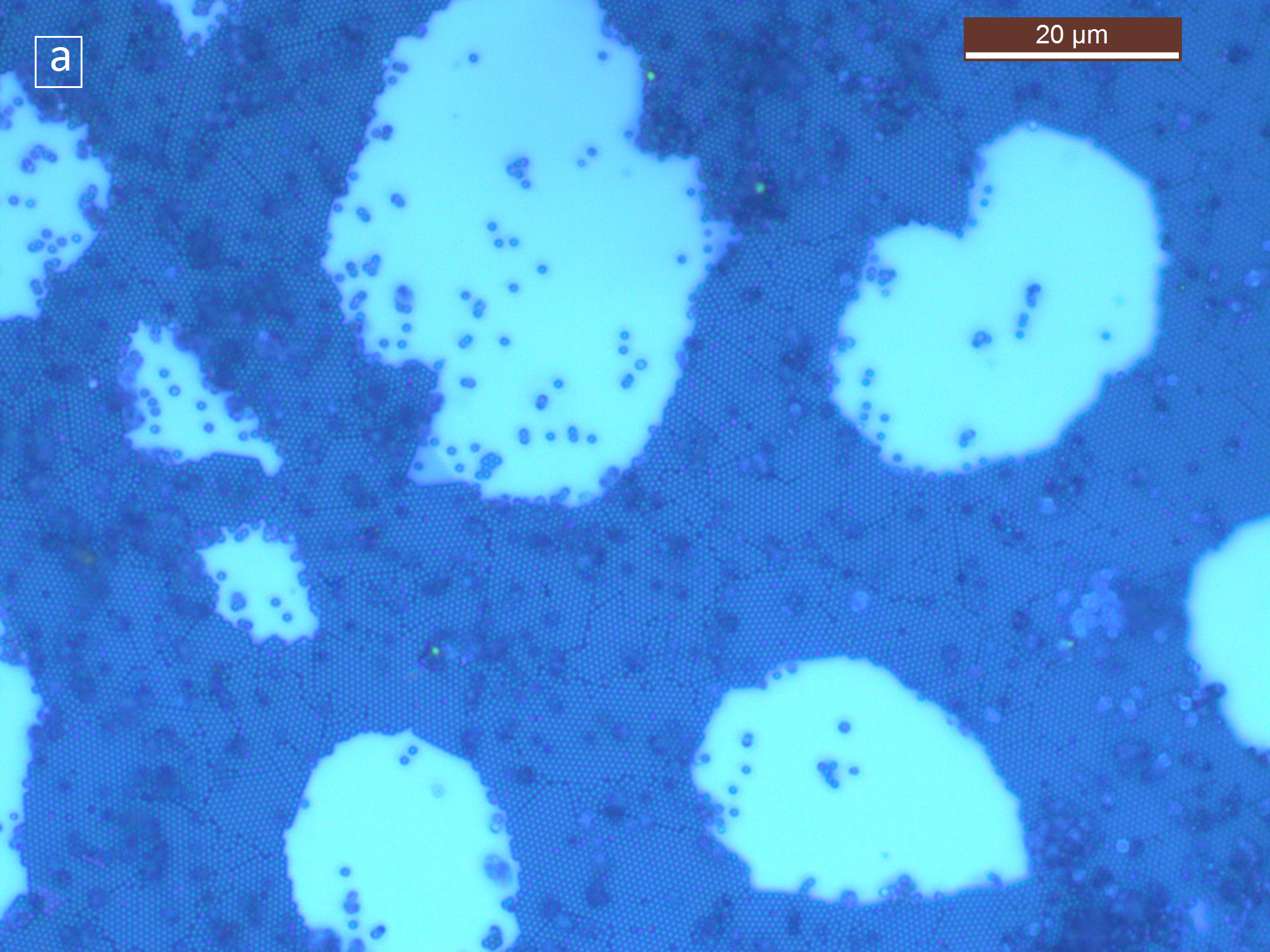

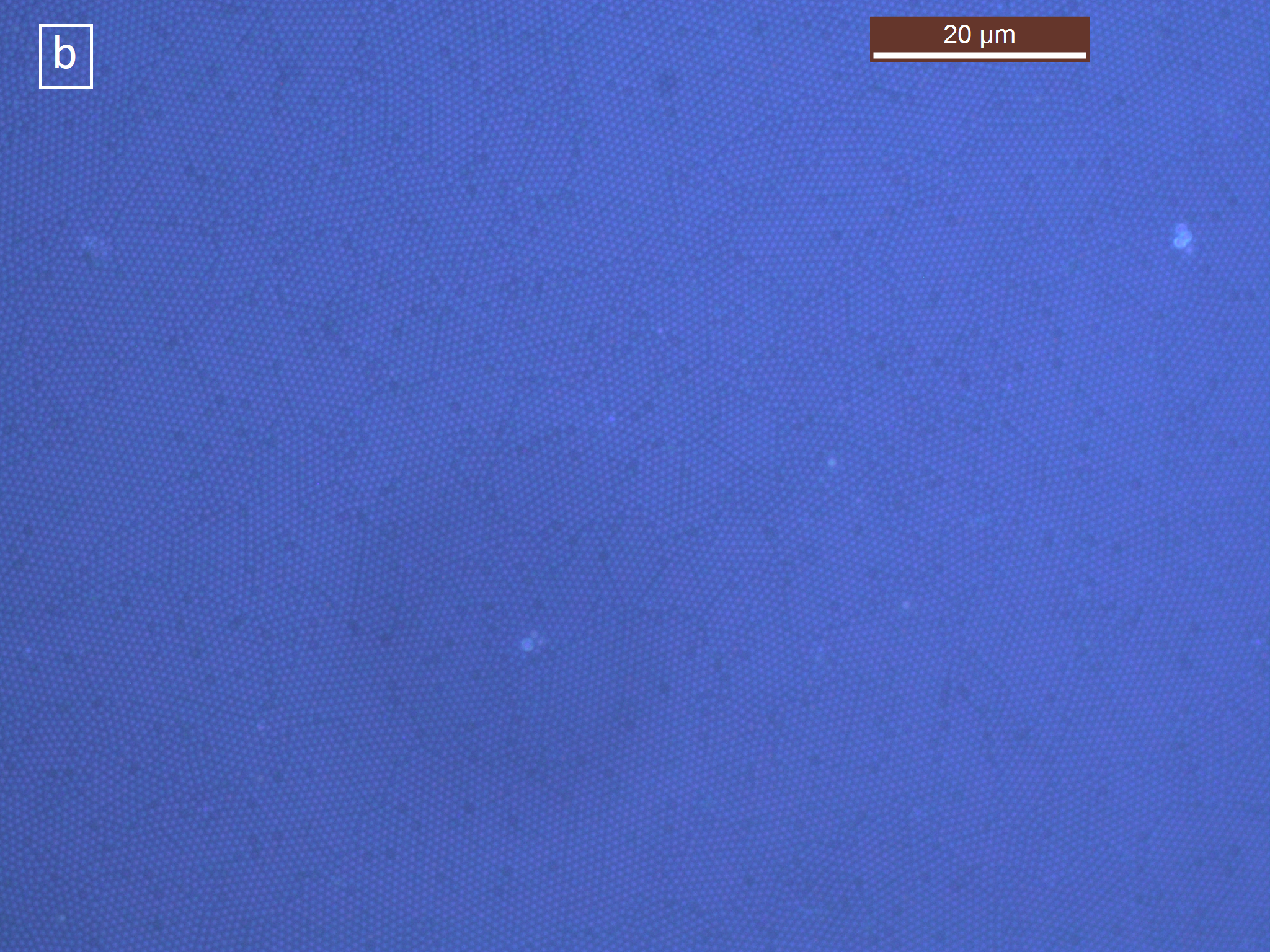


Figure S4. *Spin coated array with (a) PS particles and (b) Janus PS particles on Gold coated Silicon Wafer Substrate.*

**References:**

1. Berendsen, H. J. C., Grigera, J. R. & Straatsma, T. P. The missing term in effective pair potentials. *J. Phys. Chem.* 1987**,** 91, 6269–6271.
2. Molotilin, T. Y., Lobaskin, V. & Vinogradova, O. I. Electrophoresis of Janus particles: A molecular dynamics simulation study. *J. Chem. Phys.* 2016, 145**,** 244704.
3. Koplik, J. & Maldarelli, C. Molecular dynamics study of the translation and rotation of amphiphilic Janus nanoparticles at a vapor-liquid surface. *Phys. Rev. Fluids* 2019, 4**,** 044201.
